# Regulation of metabolic and transcriptional responses by the thyroid hormone in cellular models of murine macrophages

**DOI:** 10.3389/fimmu.2022.923727

**Published:** 2022-07-22

**Authors:** Irene López-Mateo, Diego Rodríguez-Muñoz, Juan Vladimir de La Rosa, Antonio Castrillo, Susana Alemany, Ana Aranda

**Affiliations:** ^1^ Instituto de Investigaciones Biomédicas “Alberto Sols”, Consejo Superior de Investigaciones Científicas (CSIC)-Universidad Autónoma de Madrid, Madrid, Spain; ^2^ Unidad de Biomedicina (Unidad Asociada al CSIC), Universidad de las Palmas de Gran Canaria, Las Palmas, Spain; ^3^ Centro de Investigación Biomédica en Red de Cáncer (CIBERONC), Instituto de Salud Carlos III, Madrid, Spain

**Keywords:** thyroid hormones, immortalized macrophages, primary macrophages, proliferation, signaling pathways, metabolites, transcriptome

## Abstract

Oncogene-immortalized bone marrow-derived macrophages are considered to be a good model for the study of immune cell functions, but the factors required for their survival and proliferation are still unknown. Although the effect of the thyroid hormones on global metabolic and transcriptional responses in macrophages has not yet been examined, there is increasing evidence that they could modulate macrophage functions. We show here that the thyroid hormone T3 is an absolute requirement for the growth of immortal macrophages. The hormone regulates the activity of the main signaling pathways required for proliferation and anabolic processes, including the phosphorylation of ERK and p38 MAPKs, AKT, ribosomal S6 protein, AMPK and Sirtuin-1. T3 also alters the levels of metabolites controlling transcriptional and post-transcriptional actions in macrophages, and causes widespread transcriptomic changes, up-regulating genes needed for protein synthesis and cell proliferation, while down-regulating genes involved in immune responses and endocytosis, among others. This is not observed in primary bone marrow-derived macrophages, where only p38 and AMPK activation is regulated by T3 and in which the metabolic and transcriptomic effects of the hormone are much weaker. However, the response to IFN-γ is reduced by T3 similarly in immortalized macrophages and in the primary cells, confirming previous results showing that the thyroid hormones can antagonize JAK/STAT-mediated signaling. These results provide new perspectives on the relevant pathways involved in proliferation and survival of macrophage cell culture models and on the crosstalk between the thyroid hormones and the immune system.

## Introduction

Macrophages are innate myeloid cells with a key function in health and disease. These cells perform essential functions in protecting organisms from pathogens, modulating the adaptive immune response through antigen processing and presentation. Macrophages also control tissue homeostasis, remodeling and repair, as well as the induction and resolution of inflammation (1). Macrophage polarization in response to different environmental signals is very important for an effective host defense against the different pathogens or for an adequate tissue repair. Quiescent macrophages can be activated toward an inflammatory (classical activation) phenotype by Toll-like receptor ligands and interferon-gamma (IFN-γ), producing pro inflammatory signals with antimicrobial properties, while the alternatively activated macrophages, exemplified by responses to IL-4 or IL-13, have an anti-inflammatory role and function mainly in tissue repair (2–4). Polarized macrophages not only acquire specific features, but also a specific metabolic program. Thus, proinflammatory macrophages undergo a metabolic switch towards enhanced glycolysis with suppression of the respiratory chain, while alternatively-activated macrophages show enhanced fatty acid (FA) oxidation and mitochondrial oxidative phosphorylation (OXPHOS) (5–13).

Cellular assays employed to study the mechanisms mediating immune cell activation mostly rely on the use of mouse bone marrow-derived macrophages (BMDM) grown *in vitro* in the presence of specific growth factors such as colony-stimulating factors (CSF) (14). Although this growth is limited by terminal differentiation, oncogenes can interfere with this process in growth factor-driven cultures. For example, fresh bone marrow (BM)-derived cells infected with the J2 recombinant retrovirus (carrying v-*myc* and v-*raf/mil* oncogenes) can grow as immortal cell lines (15). The target of the J2 virus is a CSF-l (or M-CSF)-responding cell and the immortalized cells are positive for monocyte/macrophage markers, indicating that the BM cells growing in response to infection with the J2 virus belong to the monocytic lineage (15–17). It has been recently reported that these immortal macrophages and the primary BMDMs display similar phenotypical, metabolic and functional characteristics, supporting their use in *in vitro* studies for the understanding of relevant pathways of immune cell function (18).

The thyroid hormones thyroxine (T4) and triiodothyronine (T3) are essential regulators of growth, development and metabolism. Most actions of T3, the most active thyroid hormone, are initiated by binding to the nuclear thyroid hormone receptors (TRs), encoded by the *TRα* and *TRβ* genes, which result in several isoforms with different expression and functions (19, 20). There is increasing evidence of a functional crosstalk between the thyroid hormones and the immune system, but our understanding of their role as modulators of macrophage functions is still limited and somewhat contradictory (21, 22). In general published studies suggest that the thyroid hormones could modulate macrophage phagocytic activity (23–27) and that elevated levels of T3 could favor a classically activated proinflammatory phenotype (26, 28, 29), although high-concentrations of T4 were also described to have an opposite effect (30). TRα is the predominant isoform in macrophages (26, 31). BMDMs from *TRα* knockout mice display basally elevated levels of proinflammatory cytokines (32, 33), but they also show reduced IL-1β and GM-CSF mRNA in response to lipopolysaccharide (LPS) (26). TRβ has an expression level several orders of magnitude lower than TRα in macrophages and therefore a functional relevance of this receptor in this cell type has not been well established (29). In addition, the thyroid hormones can modulate cytokine signaling and transcriptional effects. Significantly, we found that T3 attenuates activation of Signal Transducer and Activator of Transcription 3 (STAT-3) by LPS or IL-6 through a TR-dependent mechanism. Thus, inhibition of STAT-3 activation by T3 could also have potent regulatory functions during infection and inflammation (34).

In this work we found that T3 is required for survival and proliferation in an established model of immortalized bone marrow-derived macrophages (I-BMDM). The hormone regulates the activity of the major enzymes that regulate cellular growth and metabolism, including ERK, p38, AKT, mTORC1, AMPK and Sirtuin 1. This is accompanied by significant changes in the levels of low molecular weight and lipid metabolites, which are known to directly affect macrophage functions by controlling transcriptional and post-transcriptional events, and with widespread changes in gene expression, mainly related to stimulation of protein synthesis and cell proliferation. STAT-1 activation in response to IFN-γ is also reduced by T3 in I-BMDMs, reinforcing the concept that thyroid hormones can antagonize JAK/STAT-mediated signaling. This inhibition, as well as an inhibition of p38 and AMPK phosphorylation, is maintained in T3-treated primary quiescent macrophages (P-BMDMs). However, the metabolic and transcriptomic effects of T3 in these cells are much weaker and the enriched pathways are also different. Therefore, although oncogene-immortalized BMDMs might be a relevant model for the study of some macrophage responses, due to major differences between them and primary BMDM in response to T3, caution should be taken when using these cells to analyze functionally relevant effects of the hormone in macrophages in a physiological setting.

## Materials and methods

### Differentiation protocols of primary macrophages (P-BMDM)

Mice were maintained under pathogen-free conditions in a temperature-controlled room and a 12-h light-dark cycle in the animal facility of the Instituto de Investigaciones Biomédicas CSIC-UAM, Madrid. After euthanasia with CO_2_ of 5- to 7-week-old C57BL/6 female mice, bone marrow (BM) cells were extracted from the tibias and femurs by gentle centrifugation at 600 g x 1min. After lysis of red blood cells, 3-4 million BM cells were plated in p100 dishes in RPMI supplemented with 10% fetal bovine serum (FBS) (Gibco), penicillin (100 U/ml) (Sigma) and streptomycin (100 µg/ml) (Sigma). For BMDM cell differentiation, cultures were normally treated in this medium with 0,04 µg/ml of M-CSF for 5-7 days. Alternatively, differentiation was performed with 10% FBS depleted of thyroid hormones by treatment with AG1-X8 resin (hormone depleted medium), with 1% FBS supplemented with 2% fatty acid free bovine serum albumin (BSA) or with 10% serum replacement (SR, Gibco Life Tech) in the presence of M-CFS. Cells were differentiated in the presence or absence of 10 nM T3 as indicated. For experiments, P-BMDMs differentiated with complete 10% FCS and M-CSF were trypsinized and plated in 6-well plates (1million cells/well) in complete medium. After 5 h cells were shifted to medium supplemented with 10% depleted serum and treated in the presence and absence of 10 nM T3 for different time periods. When indicated, cells were treated with T3 alone for 36 h and/or with 5 ng/ml IFN-γ for the last 0-120 min.

### Immortalized BMDMs

Bone marrow-derived macrophages, immortalized using J2 retrovirus (I-BMDMs) have been previously described (35). I-BMDMs were plated in complete medium (containing 10% FCS) and, similarly to P-BMDMs, after 5 h the medium was changed to another containing 10% FCS depleted of thyroid hormones (hormone-depleted medium) and treatment with T3 started.

### MTS assay and cell counts

Cell viability assays were performed with colorimetric MTS kit (Abcam, ab197010). In metabolically active cells the tetrazolium compound 3-(4,5-dimethylthiazol-2-yl)-5-(3-carboxymethoxyphenyl)-2-(4-sulfophenyl)-2H-tetrazolium, in the presence of phenazine ethosulfate, produces a formazan dye that has an absorbance maximum at 490 nm in phosphate-buffered saline. Assays were performed in 96-wells plates (10.000 cells/well), measuring absorbance at 490 nm in a microplate reader at the times indicated. The number of cells was also counted manually in a standard hemocytometer Neubauer chamber under low power microscopy. Cells (aliquots of 20 μl) were stained with 0.4% Trypan Blue and total and dye excluding cells were counted to score the % of alive and dead cells.

### Flow cytometry

For cell cycle analysis, floating and adherent I-BMDMs were collected, washed, fixed and centrifuged. Pellets were stained with propidium iodide (PI) and sorted in FACScan Q4 (Becton Dickinson, Mountain View, CA) cell sorter. Percentages of cells in sub-G1, G1, S, and G2-M phases were calculated with Mod Fit software for Windows. For analysis of apoptosis, I-BMDM cells were stained with Annexin V (A1319, Life Technologies) plus 4’,6-diamidino-2-fenilindol (DAPI) and immediately subjected to flow cytometry analysis. Live cells were identified as DAPI^-^/Annexin V^-^, cells at the early apoptosis stage as DAPI^+^/Annexin V^-^, and late apoptosis stage as DAPI^+^/Annexin V^+^ cells. For determination of the % of macrophages in the primary BMDM cultures differentiated with different protocols, cells were trypsinized, centrifuged, resuspended in FACS buffer and incubated for 20 min in the dark at RT with F4/80-APC antibody. The absolute number of cells was calculated by adding 10 µl Perfect-Count microspheres (Cytognos) to the flow cytometry samples. Live cells were identified by adding 1 µl DAPI (32670, Sigma-Aldrich). Unstained cells were used as a negative control, to establish the flow cytometer voltage setting, and single-color positive controls were used to adjust compensation. Cell fluorescence was analyzed using a FACS CANTO II flow cytometer (BD), and analyzed with FACSDiva (Becton and Dickinson) or FlowJo (Tree Star) software.

### Protein extraction and western-blotting

Total proteins were extracted from cells in Ripa Buffer (50mM Hepes pH7.5, 150mM NaCl, 10% Glicerol, 1mM EGTA, 1% Triton-X100, 1% Sodium Doxicolate, 0,1% SDS). Cells were harvested and lysed in triple-detergent lysis buffer (50 mM Tris-HCl pH 8.0, 150 mM NaCl, 0.1% SDS, 1% NP-40, 0.5% sodium deoxycholate). In all cases, phosphatase and protease inhibitor cocktail tablets (Roche, Basel, Switzerland) were added. Protein lysates were mixed with Laemmli sample buffer, boiled and loaded onto 8% or 12% sodium dodecyl sulphate-polyacrylamide gel electrophoresis. Western analysis was performed using the antibodies and dilutions included in the **Supplementary Materials**.

### ^1^H-NMR spectrophotometry metabolomic analysis

For metabolomic analysis P-BMDM were obtained from 5 mice, pooled and plated in complete medium in p60 plates (3 million cells). After 5 h, cultures were shifted to DM and treated in the presence and absence of 10 nM T3 for 40 h. Cells were trypsinized, washed in PBS, counted and centrifuged. I-BMDM were inoculated at a concentration of 2 million cells/plate and the same protocol was followed. Pellets of frozen cells were shipped on dry ice to Biosfer Teslab (Reus, Spain). Cell pellets were extracted by adding 660 µl of a cold mixture of dichloromethane/methanol (2:1 v/v). The resulting suspension was vortexed and bath-sonicated for 1 min. Then 140 μl of cold MilliQ water were added, and samples were vortexed again. Organic and aqueous layers were allowed to equilibrate for 10 minutes at room temperature. Cell lysates were centrifuged (15,000 rpm, 15 min at 4 °C), and 320 µl of aqueous phase (upper layer) and 500 µl of organic phase (down layer) were collected for drying under a stream of nitrogen during 6 h. Cell pellets were resuspended in 600 μl of D_2_O and transferred to a 5mm NMR tube. H-NMR spectra were acquired in a BRUKER AVANCE III 600 operating a frequency of 600.2 MHz, at a temperature of 300 K for aqueous extracts and 286 K for lipidic extracts. Aqueous low molecular weight metabolites (LMWMs) were identified and quantified in the 1D Carr-Purcell-Meiboom-Gill (CPMG) spectra using an adaptation of Dolphin (36). Each metabolite was identified by checking for all its resonances along the spectra, and then quantified using line–shape fitting methods on one of its signals (37). Quantification of lipid signals in (1)H-NMR spectra was carried out with LipSpin (38) an in-house software based on Matlab. Resonance assignments were done on the basis of literature values (39). The results obtained were normalized by the number of cells in each sample.

### Real-time quantitative RT-PCR

RNA was extracted with TRI reagent Solution (AM9738, Invitrogen AM9738) and quantified in Nanodrop ND-100 (Thermo Scientific). One microgram of RNA was treated with RNase-Free DNase (79254, Qiagen 79254) and cDNA synthesis was performed with iScript cDNA Synthesis Kit (170-8891, BIO-RAD 170-8891) with oligo-dT and Random hexamers primers in a reaction protocol of 5 minutes at 25°C, 30 minutes at 42°C and 5 minutes at 85°C. qPCR was performed with Fast SYBR Green Master Mix (4385612, Applied Biosystems) on a Stratagene Mx3005P Real-Time PCR machine. The sequences of the oligonucleotides used are listed in the Supplementary Material. The thermal cycling conditions used were: activation of the polymerase 95°C for 20 seconds, 40 cycles of denaturation at 95°C for 3 seconds and annealing and extend step at 60°C for 30 seconds. Data analysis was done using the comparative cycle threshold (CT) method and transcripts were normalized to the internal control 18S RNA.

### RNA sequencing

For transcriptomic analysis, BM cells from five independent mice were pooled and differentiated for 5 days in complete medium with M-CSF. P-BMDM were then split and plated in p60 dishes (3 million cells/plate) in complete medium. After 5 h, cultures were shifted to thyroid hormone depleted medium and treated in the presence and absence of 10 nM T3 for 40 h. Transcriptomic analysis was carried out in triplicate cultures of P-BMDM and of I-BMDM cultured under the same conditions. RNA integrity was checked on an Agilent 2100 Bioanalyzer. RNA sequencing (RNAseq) was then carried out by BGI Genomics (Huada Gene, Wuhan, China) (project#F21FTSEUHT0497 and F21FTSEUHT0498). Briefly, total RNA was fragmented into short fragments and mRNA was enriched using oligo (dT) magnetic beads, followed by cDNA synthesis. Double-stranded cDNA was purified and enriched by PCR amplification, after which the library products were sequenced. The gene ontology of biological processes (GO-p) and Kyoto Encyclopedia of Genes and Genomes (KEEG) bioinformatics analysis were performed using BGI propietary Dr. TOM software, an in-house customized data mining system of the BGI (https://biosys.bgi.com). The software performed the enrichment analysis, calculated the Pvalue, and the Qvalue was obtained by PDR correction of Pvalue. A Qvalue < 0.05 was regarded as a significant enrichment. This software also uses the official software package for the analysis of gene set enrichment analysis (GSEA) (40, 41).

### Statistical analysis

Two-tailed Student’s t-tests were used for comparisons between two groups. A chi squared test was performed to evaluate differences of categorical variables. One-way ANOVA with *post*-*hoc Tukey* test was used to compare all pairs of columns from at least three different groups. The results are always expressed as means ± SD. P values <0.05 were considered statistically significant. The significance is indicated in the Figures as *p < 0.05, **p < 0.01, and ***p < 0.001. Statistics were performed with GraphPad Prism 7.0 software.

## Results

### T3 is required for proliferation and survival of I-BMDM

**Figure 1:**
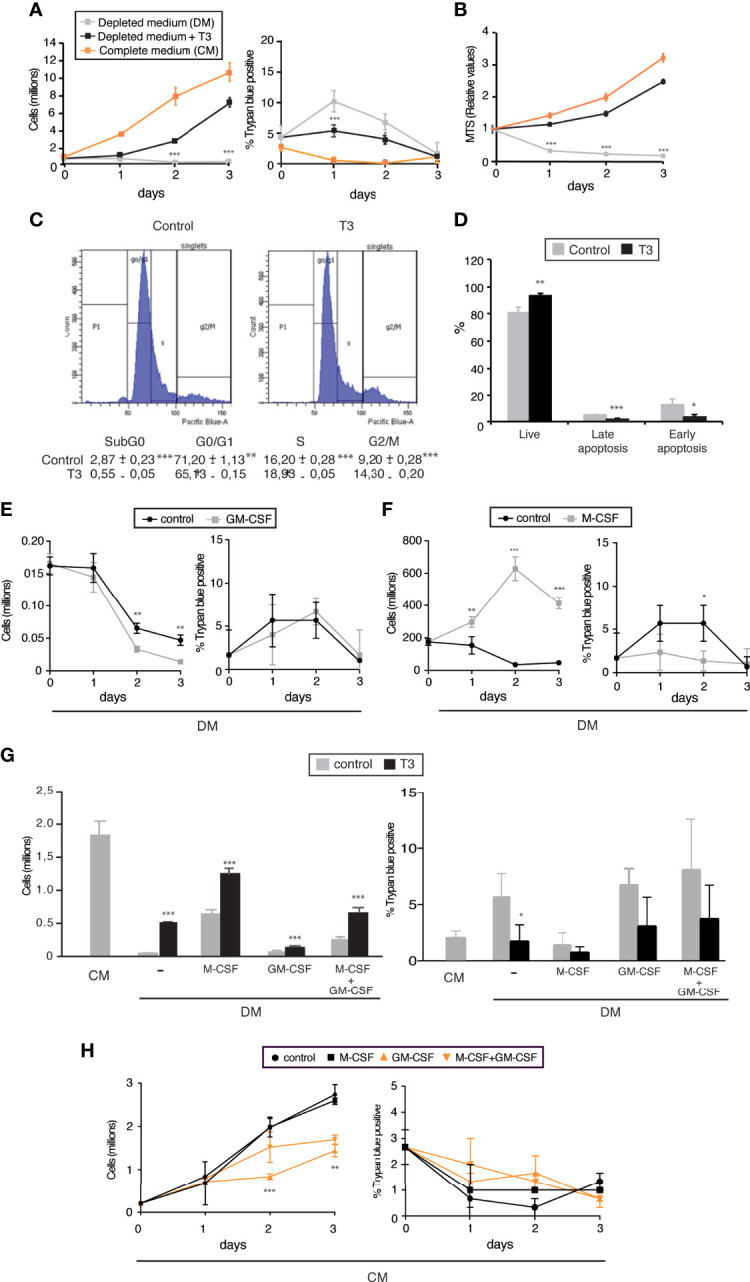
T3 is required for proliferation and viability of J2-immortalized macrophages. **(A)** Immortalized bone marrow derived macrophages (I-BMDM) were plated in 6-well plates in complete medium containing 10%FCS (CM) and after 5 h were either maintained in the same medium or shifted to medium supplemented with 10% thyroid hormone-depleted medium (DM) in the absence or presence of 10 nM T3 (n=9). Total cell number (left panel) as well as the percentage of Trypan blue positive cells (right panel) were counted manually in a Neubauer chamber under low power microscopy at the times indicated. **(B)** MTS [3-(4,5-dimethylthiazol-2-yl)-5-(3-carboxymethoxyphenyl)-2-(4-sulfophenyl)-2H-tetrazolium] levels determined in the same experimental groups in 96-well plates (n=9). **(C)** Cell cycle analysis by flow cytometry of I-BMDM cultured for 48 h in depleted medium in the absence (Control) and presence of T3. A representative plot as well as the % of cells in the different stages of the cell cycle (n=3) are shown. **(D)** The percentage of total cells that were PI-/Annexin V- (alive cells); PI+/Annexin V- (early apoptosis) and PI+/Annexin V+ (late apoptosis) was determined by flow cytometry in I-BMDM grown in depleted medium in the absence and presence of T3 for 48 h (n=3). **(E)** I-BMDM were cultured in 24-well plates in complete medium, shifted to depleted medium (DM) after 5 h and incubated for the times indicated in the absence (control) and presence of M-CSF (40 ng/ml). The number of cells (left panel) as well as the percentage of Trypan blue positive cells (right panel) were scored manually under the microscope (n=3). **(F)** Cells were cultured as in E with and without GM-CSF (20 ng/ml) and the number of cells counted at the times indicated (n=3). **(G)** I-BMDM were cultured in complete medium (CM) or shifted after 5 h to hormone-depleted medium (DM) supplemented with M-CSF and/or GM-CSF in the presence and absence of T3. The number of total (left panel) and Trypan blue positive cells (right panel) was scored 48h later (n=6). **(H)** Cells were plated in complete medium (CM) and incubated in the same medium with M-CSF and/or GM-CSF. The total number of cells (left panel, as well as the number of Trypan blue positive cells were determined at the times indicated. Data are means ± SD. ^*^p < 0.05, ^**^p < 0.01, ^***^p < 0.001.

Counts of immortalized bone marrow derived macrophages (I-BDMD) show that cells cultured in complete medium with 10% FBS grow rapidly, with only a minor percentage of Trypan Blue positive cells. In contrast, I-BDMD cultured in medium supplemented with 10% FBS depleted of thyroid hormone (depleted medium) were unable to proliferate and show a transient increase in permeability to the cell dye, which was never higher than 10%. However, when 10 nM T3 was added back to the depleted medium I-BDMD regained the capacity to grow and the percentage of Trypan Blue positive cells was again very low (**Figure 1A**). MTS cell viability assays showed a similar pattern. The number of metabolically active cells was markedly reduced in a time-dependent manner in the absence of thyroid hormone, while incubation with T3 of cells cultured in depleted medium increased MTS values close to those of cultures grown in complete medium (**Figure 1B**). These data suggest that T3 increases cell proliferation and/or reduces cell death. Thus, we next analyzed the cell cycle of I-BDMD grown in hormone-depleted medium in the absence (control) and in the presence of T3 for 48h. As shown in **Figure 1C**, in comparison with control cultures, T3-treated cultures showed a reduced percentage of I-BDMD arrested in G0/G1, and a concomitant increase in the percentage of cells in S- and G2/M phases. In addition, the percentage of sub-G0/G1 cells was lower in T3-treated cultures, suggesting that the hormone besides increasing proliferation could also reduce I-BDMD death. An increased rate of apoptosis could contribute to the low number of cells cultured in depleted medium in the absence of T3. To analyze this possibility, the frequency of apoptotic cells was determined by measuring DAPI and Annexin V staining by flow cytometry. In T3-treated cells a detectable increase in the percentage of live (DAPI^-^/Annexin V^-^) I-MBDM, from 80% to more than 90% was observed, corresponding to a reduction in both late apoptotic (DAPI^+^/Annexin V^+^) and early apoptotic (DAPI^-^/Annexin V^+^) cells (**Figure 1D**). These data indicate that the thyroid hormones are required for proliferation and survival of immortalized macrophages. As reported for normal macrophages (29), *TRβ* mRNA levels were several orders of magnitude lower than *TRα* mRNA levels in both control and T3-treated I-BDMD (**Figure S1**), suggesting that the TRα isoform would be responsible for the actions of T3.

Since CSFs are important modulators of macrophage proliferation, we next tested the effect of M-CSF and GM-CSF on the growth of I-BMDM. While M-CSF strongly increased proliferation of the immortalized cells grown in depleted medium (**Figure 1E**), unexpectedly GM-CSF had an opposite effect and after 48h reduced the number of I-BMDM. This reduction was not due to an increase in the percentage of Trypan Blue positive cells, which was low under this condition (**Figure1F**). Furthermore M-CSF had an additive effect with T3, reaching in depleted medium up to 70% of the number of cells observed in complete medium after 48 h of treatment. However, GM-CSF was able to partially block the effect of T3 and/or M-CSF (**Figure 1G**). This again occurred without a significant decrease in cell viability. Reduced proliferation in the presence of GM-CSF without increased cell death was also observed when the immortalized macrophages were cultured in complete medium containing endogenous thyroid hormones, both in the absence and presence of M-CSF, which in this medium did not increase cell number (**Figure 1H**).

### Effect of T3 in the generation of primary BMDM

**Figure 2:**
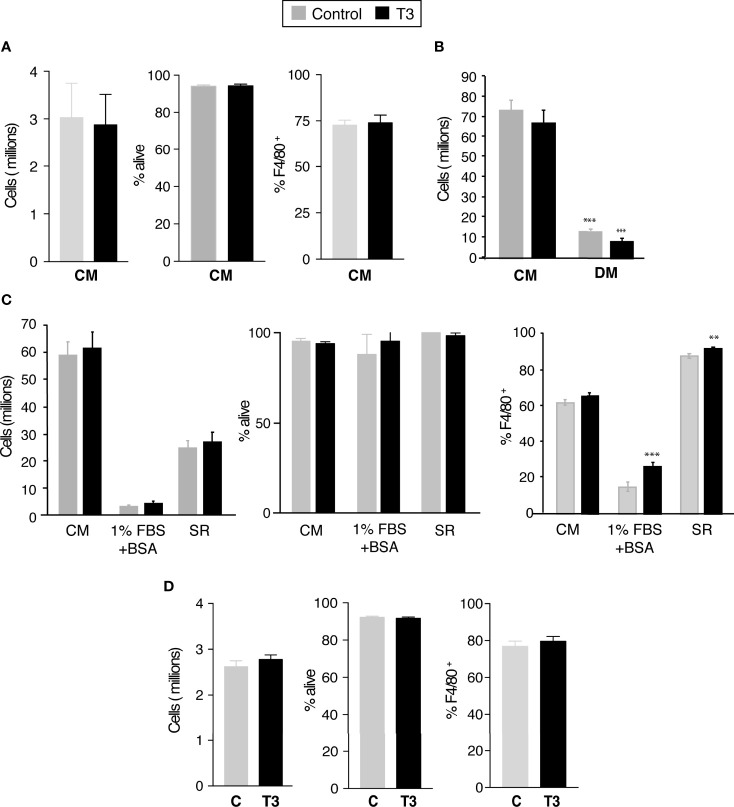
T3 is not required for the generation of primary bone marrow derived macrophages. **(A)** Bone marrow derived cells were differentiated during 5 days in complete medium (CM, 10% FBS supplemented with 40 ng/ml M-CFS) in the presence (control) and absence of 10 nM T3. Total number of cells, cell viability and % of F4/80^+^ cells was determined by flow cytometry (n=12). **(B)** Bone marrow derived cells were differentiated during 5 days in complete medium (CM) or in hormone-depleted medium (DM, 10% thyroid hormone depleted FBS) supplemented with 40 ng/ml M-CFS) in the presence and absence of 10 nM T3. Total number of cells was determined by flow cytometry (n=6). **(C)** Bone marrow cells were plated in complete medium and after an overnight incubation were differentiated in the same medium (CM), in medium containing 1%FBS plus 2% BSA or in 10% serum replacement medium (SR) for 5 days. In all cases medium was supplemented with M-CSF and cells were cultured in the presence and absence of T3. Total number of cells, cell viability and % of F4/80^+^ cells was determined by flow cytometry (n=3). **(D)** Cells differentiated in complete medium and M-CSF for 5 days were shifted to hormone-depleted medium. Cell number, viability and % of F4/80^+^ cells were determined 48 h later in untreated and T3-treated cells (n=12). Data are means ± SD. ^**^p < 0.01, ^***^p < 0.001.

High concentrations of T3 have been proposed to have a negative effect in the differentiation of bone marrow-derived monocytes into unpolarized macrophages (28). However, when bone marrow cells were differentiated in complete medium containing M-CSF in the presence of 10 nM T3 during 7 days, both the number of adherent viable cells and the % of cells expressing the F4/80^+^ macrophage marker were similar to those obtained in the absence of exogenously added T3 (**Figure 2A**). In addition, with the exception of a moderate reduction in the mRNA levels of *IL12a*, mRNA levels encoding for genes selectively expressed by pro-inflammatory or alternatively-activated macrophages were not altered in cells differentiated in the presence of T3 (**Figure S2**).

There was the possibility that endogenous T3 in the FBS could mask the effect of the hormone on bone marrow monocyte differentiation into macrophages. Therefore, we next tried to differentiate the bone marrow cells in DM (**Figure 2B**). The yield of cells, as well as the percentage of F4/80^+^ cells, was strongly decreased under these conditions, but the presence of T3 during the differentiation process did not reverse this reduction, indicating that treatment of the FBS removes other compound/s, besides the thyroid hormones, necessary for BM cell proliferation and differentiation into macrophages. Addition of T3 to differentiating BM cells in which 10% FBS was substituted by 1%FBS+2% BSA did not increase the extremely low number of cells in the cultures, although it caused a moderate but significant increase in the % of cells expressing the macrophage marker. Moreover, when cells were differentiated with a serum replacement (SR) medium, which does not contain thyroid hormones, the yield of cells was approximately 40% of that obtained in the complete medium and T3 did not increase this value, although it caused a slight increase in the % of the F4/80^+^ macrophage cell population (**Figure 2C**). Collectively, these results indicate that physiological concentrations of T3 do not impair proliferation or differentiation of bone marrow-derived monocytes into macrophages, but could rather have a moderate positive effect in this process. We next analyzed whether T3 could affect post-mitotic primary macrophages (P-BMDM) differentiated in complete medium and then shifted to DM. As shown in **Figure 2D**, incubation with T3 for 48 h did not influence macrophage cell number, viability, or overall percentage of F4/80^+^ cells.

### T3 regulates expression of pro- and anti-inflammatory genes

Previous work has shown that the thyroid hormone through binding to TRα can regulate mRNA levels of *GM-CSF* (26), the M2 marker *Arginase 1* (29), and the proinflammatory cytokine *TNFa* (32, 33) in macrophages. As shown in **Figure S3**, T3 increased significantly transcript levels not only of *GM-CSF*, but also of *M-CSF* in I-BMDM, suggesting the existence of autocrine mechanism in the regulation of proliferation by the hormone. The levels of mRNA of the pro-inflammatory cytokine *TNFα*, as well as of *HIF-1α*, a critical regulator of macrophage activation that leads to expression of inflammatory cytokines and chemokines (42) were also up-regulated by T3. However, T3 also induced expression of *Arginase 1* mRNA, a marker of immunomodulatory macrophages. Only transcripts for *GM-CSF* and *Arginase 1* showed a tendency to be increased by T3 in BMDM, although this increase was not statistically significant.

### Influence of T3 on the activity of key signaling pathways regulating proliferation, viability and metabolism

**Figure 3:**
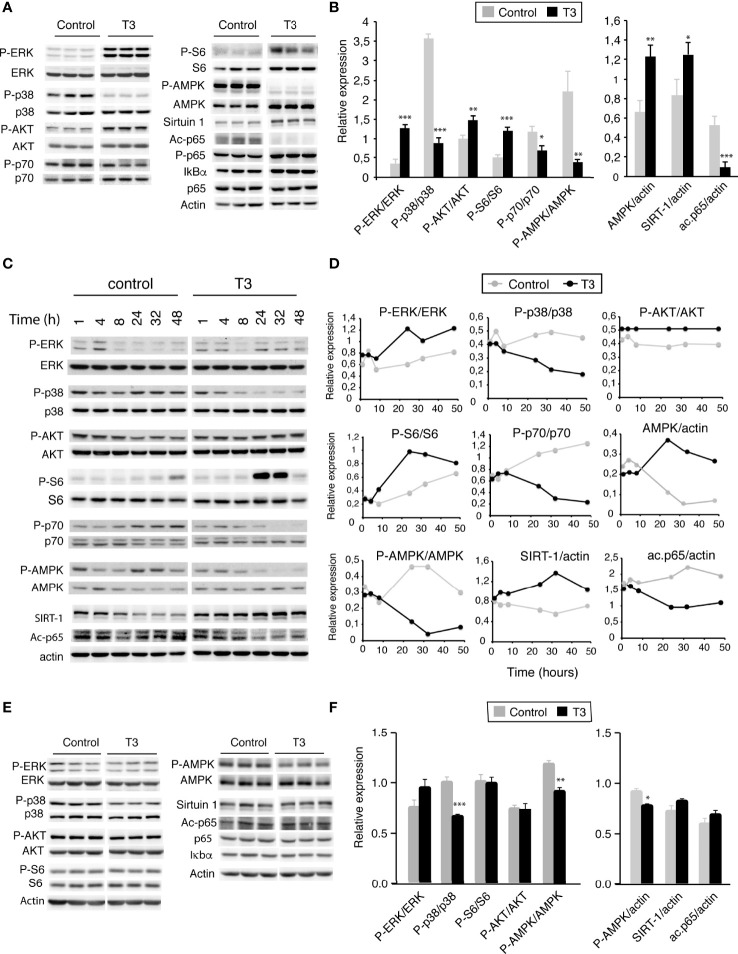
T3 alters activity of key signaling pathways that control metabolism, viability and proliferation. **(A)** I-BMDM plated in complete medium were shifted to hormone-depleted medium after 5 h and incubated with and without 10 nm T3 for 48h. Representative western blot analysis with the indicated antibodies are shown. **(B)** Quantification from 6 independent experiments showing relative values of phosphorylated versus total proteins or relative to actin levels used as a loading control. **(C)** I-BMDM plated in complete medium were shifted to depleted medium and after 5 h incubated with and without 10 nM T3 for indicated time periods, ranging between 1 and 48h. Representative western blots with the indicated antibodies are shown. **(D)** Quantification of western blots from two independent experiments with variations lower than 20%. **(E)** Primary cultures of BMDM (P-BMDM) differentiated for 5 days in complete medium were shifted to hormone-depleted medium and incubated in the absence (control) and presence of 10 nM T3 for 48 h. Representative western blot analysis with the indicated antibodies are shown. **(F)** Quantification of the western blots from 6 independent experiments in control and T3-treated cells showing relative values of phosphorylated versus total proteins or values relative to actin levels used as a loading control. Data are means ± SD. *p < 0.05, ^**^p < 0.01, ^***^p < 0.001.

Mitogen-activated protein kinases (MAPK) are crucial to sustain proliferation and to generate immune responses (43). Among the various MAPK, extracellular signal-regulated kinase ERK1/2 has been shown to be a key regulator in cell proliferation and the prevention of apoptosis (44). In agreement with this, treatment of I-BMDM with T3 for 36 h strongly increased ERK1/2 phosphorylation, without altering total ERK levels (**Figures 3A, B**). This was opposite to the effect of the hormone on activation of the stress p38 MAPK that coordinates cell survival and cell death, which was strongly reduced by T3. In addition, T3 induced AKT phosphorylation and activation of its downstream target mammalian target of rapamycin complex 1 (mTORC1), which controls major anabolic processes (45). Despite the finding that the phosphorylation of the ribosomal protein p70 S6 kinase (S6K) was reduced in T3-treated cells, the hormone increased phosphorylation of the 40S ribosomal protein S6, a crucial downstream effector of this pathway in protein translation. AMP-activated protein kinase (AMPK) is another important metabolic enzyme (46). Interestingly, incubation of I-BMDM with T3 increases total AMPKα levels (**Figures 3A, B**), as well as its mRNA (**Figure S4**), but strongly inhibits Thr172 phosphorylation and therefore its kinase activity (47). T3-treatment also caused a significant increase in the levels of the NAD^+^-dependent lysine deacetylase Sirtuin-1. This occurred by a post-transcriptional mechanism as Sirtuin-1 mRNA levels were not affected by T3 (**Figure S4**). Sirtuin-1 deacetylates the p65 component of the nuclear factor κB (NF-κB) pathway (48) and, in concert with the increased Sirtuin-1 levels, T3 strongly reduced p65 acetylation in I-BMDM without altering total p65 levels and causing minor increases of p65 phosphorylation or inhibitory IκBα levels (**Figures 3A, B**). Because T3 could induce regulation of genes which could affect these signaling pathways, the effect of T3 on their activity was also examined at shorter time points. The activation state of the analyzed signaling pathways in untreated and T3-treated cells was rather similar between 1 and 8 hours, and differences appeared after 24 hours of incubation with the hormone being maximal in most cases after 32-48 h (**Figures 3C, D**), suggesting that regulation of these pathways by T3 is indeed a secondary effect.

When the same pathways were analyzed in post-mitotic unpolarized P-BMDM, changes in ERK1/2, AKT or S6 phosphorylation were not observed after T3 treatment, although p38 phosphorylation was reduced. The hormone also decreased AMPKα phosphorylation, without changes in total protein or mRNA levels, while Sirtuin-1 and its target acetyl p65 were not affected (**Figures 3E, F**)

### Metabolic profile in T3-treated macrophages

**Figure 4:**
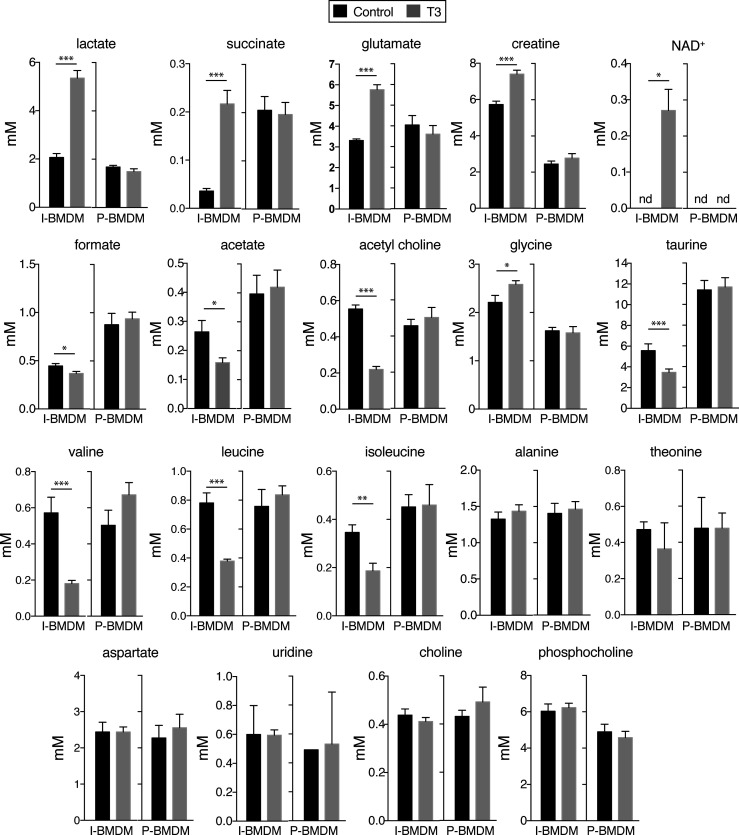
T3 regulates the levels of low molecular weight metabolites in immortalized but not in primary macrophages. Primary BMDM (P-BMDM) were differentiated in complete medium, shifted to hormone-depleted medium and incubated with (n=6) and without (n=6) 10 nM T3 for 48 h. Immortalized BMDM (I-BMDM) were also incubated without (n=5) or with T3 (n=6) in hormone-depleted medium for the same time period. Intracellular concentrations (mM) of the indicated metabolites, corrected by cell number, were determined in I-BMDM and P-BMDM by nuclear magnetic resonance spectroscopy. Data are means ± SD. ^*^p < 0.05, ^**^p < 0.01, ^***^p < 0.001.

**Figure 5:**
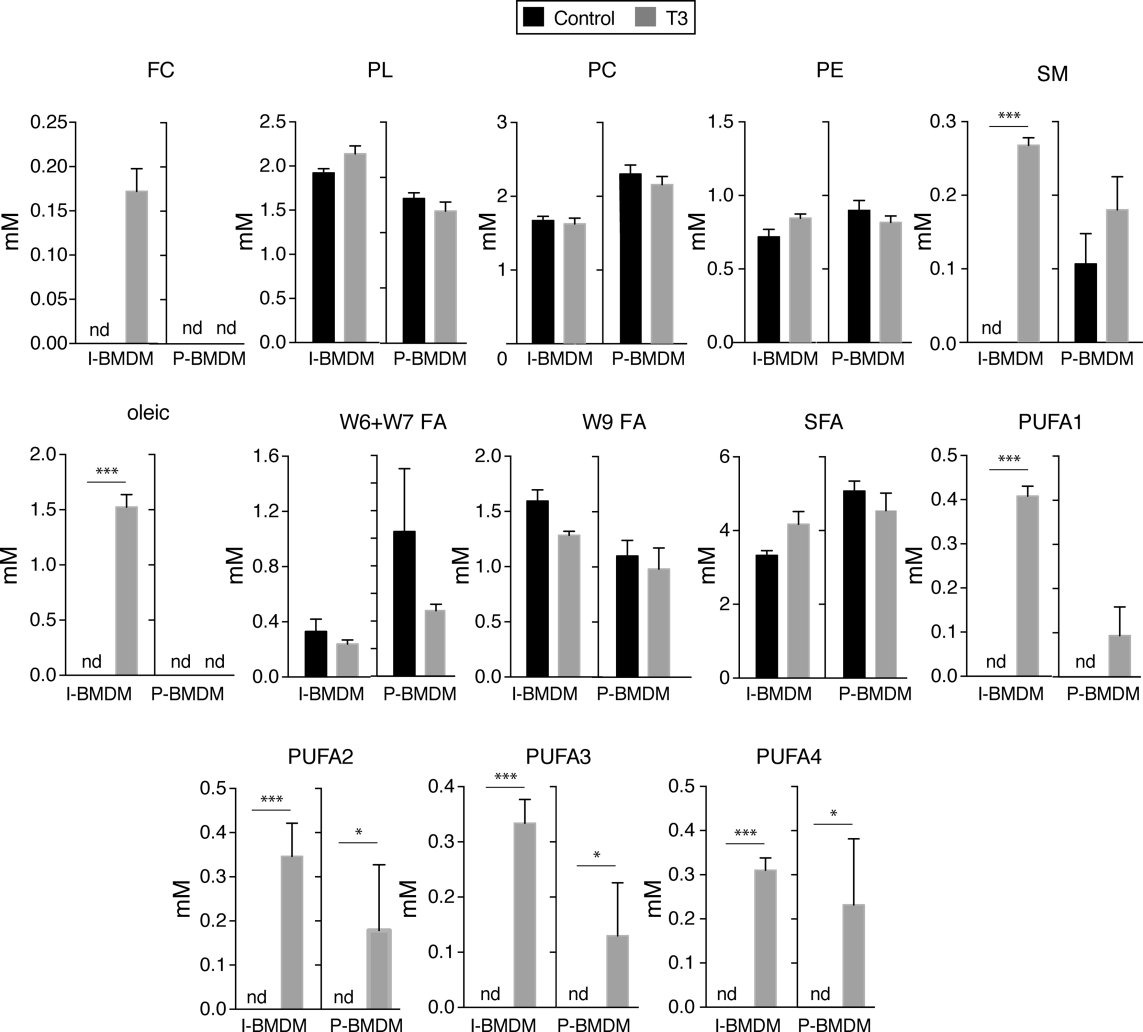
T3 increases the levels of polyunsaturated fatty acids in immortalized and primary macrophages. Concentration of lipid metabolites in the same cells of **Figure 4** are shown. FC, free cholesterol, PL, phospholipids, PC, phosphatidylcholine, PE, phosphatidylethanolamine, SM, sphingomyelin, FA, fatty acids, SFA, saturated fatty acids, PUFA, polyunsaturated fatty acids. A chi squared test was performed to evaluate a differential presence of lipid families between control and T3-treated cells when metabolites were not detected (nd) in one group. *p < 0.05; ***p < 0.001.

**Figure 6:**
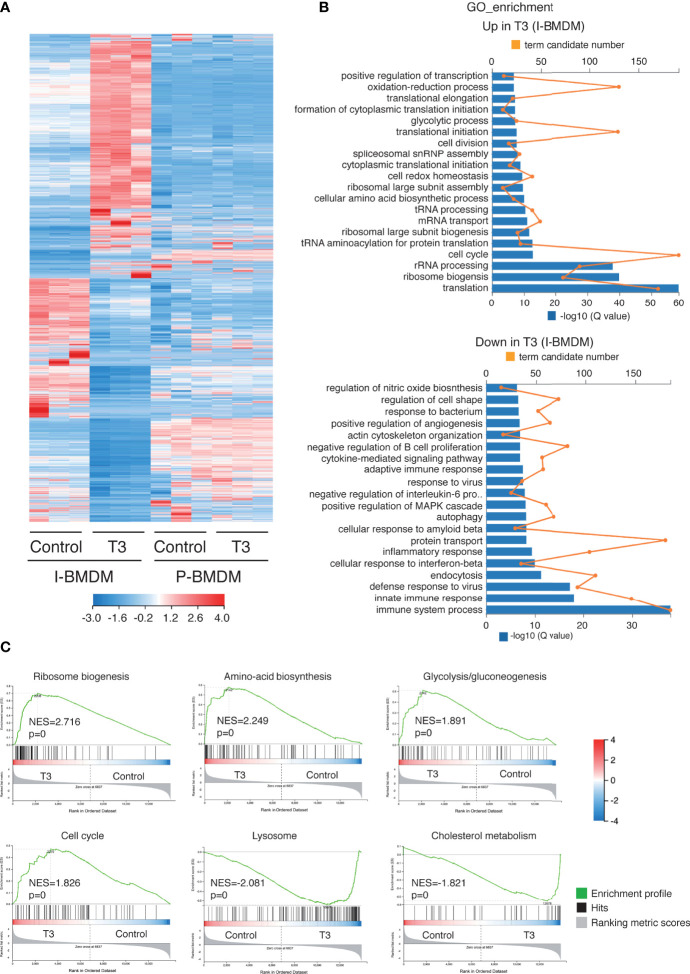
T3 induces expression of genes involved in cell proliferation and protein translation in immortalized macrophages. **(A)** Heatmap showing differential expression of transcripts between immortalized (I-BMDM) and primary (P-BDMD) cells. P-BMDM, differentiated in complete medium supplemented with M-CSF, were shifted to thyroid hormone depleted medium and cultured in absence (control) and presence of 10 nM T3 for 2 days. I-BMDM were treated under the same conditions. **(B)** Top overrepresented gene ontology (GO) categories with significant transcripts enrichment (upper panel) or reduction (lower panel) in the T3-treated I-BMBM with respect to the untreated cells. Both the term candidate transcript number (in orange) and the Q value (in blue bars) are depicted in the plots. **(C)** Enrichment plots from Gene Set Enrichment analysis (GSEA) based in KEEG pathway database showing the top over-represented hallmarks in up-regulated and down-regulated genes in T3-treated I-BMDM. The normalized enrichment scores (NES) as well as the nominal *p* values are shown.

In order to analyze whether changes in activity of signaling pathways correlated with metabolic changes, we next studied the levels of aqueous low molecular weight and lipid metabolites by nuclear magnetic resonance spectroscopy in untreated and T3-treated macrophages in DM. Incubation with T3 of I-BMDM, but not of P-BMDM, caused drastic changes in the levels of many of the detected aqueous low molecular weight metabolites (**Figure 4**). Thus, T3 increased significantly the intracellular concentrations of lactate, succinate, glutamate, creatine, and NAD^+^, while reducing the levels of formate, acetate and acetylcholine. Furthermore, with the exception of glycine, the amounts of the other detected aminoacids, including taurine, valine, isoleucine and leucine, were reduced in I-BMDM after T3 treatment. On the other hand, lipidomic analysis in I-BMDM (**Figure 5**), revealed that T3 increased the levels of free cholesterol, sphingomyelin and oleic acid. Intracellular levels of the different types of polyunsaturated fatty acids (PUFAs) were also higher upon incubation with T3 not only in immortalized but also in primary BMDM, while the levels of other fatty acids were not altered (**Figure 6**).

### Transcriptomic changes in T3-treated macrophages

To further understand the mechanism by which T3 acts in macrophages, RNA-seq was performed to profile genome-wide gene expression in immortalized and primary BMDM. T3 caused a widespread change in the transcriptome of I-BMDM (**Figure 6A**) with 3082 transcripts up-regulated and 3402 transcripts down-regulated (**Table S1**). Transcriptomic analysis confirmed up-regulation of the genes shown in **Figure S3**. Functional annotation of the up-regulated transcripts showed that the most enriched Gene Ontology (GO) of biological processes terms were related to protein synthesis and the cell cycle, consistent with the enhanced proliferation observed in T3-treated cells, while immune responses and endocytosis, among others, were enriched in the down-regulated GO terms among the T3-repressed transcripts (**Figure 6B**). Furthermore, ranked gene set enrichment analysis (GSEA) revealed that the genes up-regulated by T3 treatment of I-BMDM cells were significantly enriched in the Hallmark set of ribosome biogenesis. Other top scoring gene sets enriched in the up-regulated genes were aminoacid biosynthesis, glycolysis/gluconeogenesis and cell cycle, in agreement with the GO analysis, while lysosome and cholesterol metabolism hallmarks were found in the down-regulated genes (**Figure 6C**). RNA-seq analysis also clearly showed important transcriptomic changes in immortalized macrophages vs their primary counterparts with loss of hematopoietic and immune system processes with gain of translation and cell cycle processes (**Figure S5**).

**Figure 7:**
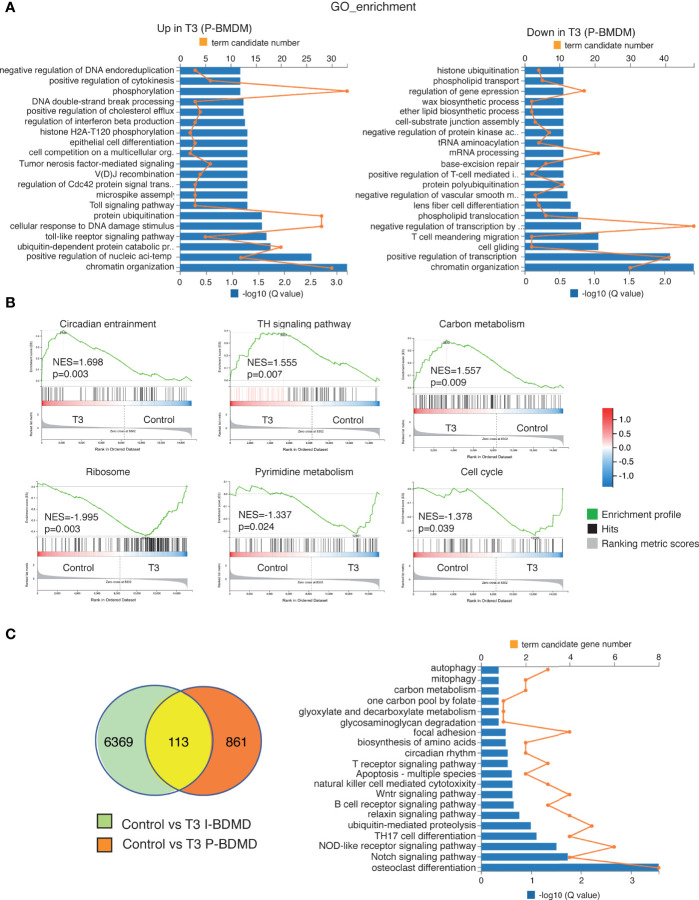
Transcriptional profiling of T3-treated primary macrophages. **(A)** Gene ontology (GO) categories with significant enrichment (left panel) or reduction (right panel) in the transcripts of T3-treated primary cultures of macrophages (P-BMDM) with respect to the untreated cells. Both the term candidate transcript number (in orange) and the Q value (in blue bars) are represented. **(B)** Enrichment plots from Gene Set Enrichment analysis (GSEA) based in the KEEG pathway database showing the top over-represented hallmarks in up-regulated and down-regulated genes in T3-treated P-BMDM. The normalized enrichment scores (NES) as well as the nominal *p* values are shown. **(C)** Venn diagram of the intersection between transcripts regulated by T3 in both I-BDMD and P-BMDM (left) and GO categories of the overlapping transcripts regulated by T3 based in *q* values (right).

As observed in the heatmap (**Figure 6A**), less transcripts were altered after T3 treatment in P-BMDM than in I-BMBM and the changes were also weaker. The list of T3 up- and down-regulated transcripts in P-BMDM is shown in **Table S2**. In addition, the GO terms regulated by T3 in P-BMDM (**Figure 7A**), were different from those found in I-BMDM, with chromatin organization and transcriptional regulation being predominant in both up- and down-regulated transcripts. GSEA analysis showed that the most enriched set of genes were also different. Unexpectedly, although no changes in cell number were observed (**Figure 2**), ribosome and cell cycle were among the T3 down-regulated terms in P-BMDM (**Figure 7B**). The Venn diagram showed that only 113 overlapping transcripts were regulated by T3 in both immortal and primary BMDM (**Figure 7C**) of which 50 showed the same trend of expression (**Table S3**), with changes in different signaling pathways being the main GO terms (**Figure 7C**).

### T3 inhibits JAK/STAT activation in response to IFN-γ

**Figure 8:**
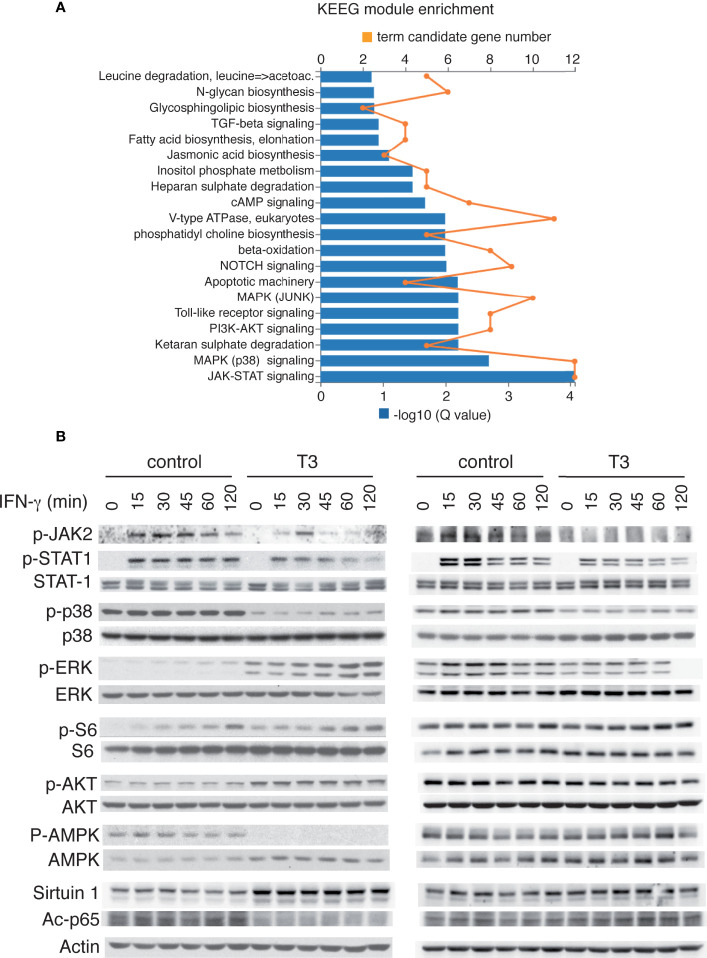
T3 inhibits JAK/STAT activation by IFNγ in immortalized and primary bone marrow-derived macrophages. **(A)** KEEG module enrichment in transcripts down-regulated by T3 in I-BMBM, showing that JAK/STAT and p38 MAPK modules are the two most down-regulated terms. Both the term candidate transcript number (in orange) and the Q value (in blue bars) are depicted. **(B)** I-BMDM (left) and P-BMDM (right) plated in complete medium were shifted to hormone-depleted medium and after 5 h incubated with and without 10 nM T3 for 40 h and with 5 ng/ml IFN-γ for the indicated time points. Representative western blots with the indicated antibodies are shown.

KEEG module pathway analysis (49) showed that the most down-regulated term in T3-treated I-BMDM was the JAK-STAT module (M00684), followed by the p38 module (**Figure 8A**). We then analyzed by western blot the response to IFN-γ in cells pre-treated or not with T3 (**Figure 8B**). As, expected, IFN-γ caused JAK-2 and STAT-1 phosphorylation, which was already maximal after 15 min, and this response was significantly blunted both in immortal and primary T3-treated macrophages. IFN-γ did not alter p38 phosphorylation that, in agreement with the results shown in **Figure 3**, was basally reduced by T3 in both cellular models, but caused the expected induction of ERK phosphorylation in P-BMDM (50), and this induction was also reduced in cells pre-treated with the hormone. This was not observed in I-BMDM where this signaling pathway was constitutively induced by T3 treatment. The cytokine also increased S6 phosphorylation in I-BMDM while the activity of AKT, AMPK or Sirtuin-1 was not affected by IFN-γ, showing only the effects of T3 already observed basally in **Figure 3**.

## Discussion

Immortalization of murine bone marrow cells by retroviral infection with the J2 oncogene, in the absence of a specific growth factor supplement, induces the selective proliferation of monocytic cells, allowing the generation of immortal cells with functional features of primary macrophages (15), which can induce formation of tumors in newborn mice (51). v-*raf* and v-*myc* might affect different components of growth regulation, as, for example, commitment (v-*myc*) and cell cycle progression (v-*raf*) (52). J2-immortalized macrophages grow rapidly in medium supplemented with fetal calf-serum in the absence of exogenous growth factors. However, these immortal cells are unable to grow when the serum has been treated with an anion exchange resin, suggesting a complementation between the viral oncogenes and other factors present in the serum. This treatment is the standard method for depleting the serum of thyroid hormones and the finding that adding back T3 to the depleted medium recovers growth to a large extent, demonstrates that this hormone is a key factor for the proliferation of immortalized macrophages. This action is most likely mediated by binding of T3 to TRα. This isoform is the major receptor in normal macrophages (29) and this feature is conserved upon immortalization. The thyroid hormones might have divergent effects on cell proliferation and transformation depending on the cell type, the cellular context, the transformation status or the expressed TR isoform (20, 53). With few exceptions, oncogene-immortalized or tumor cell lines are not responsive to the thyroid hormones and these hormones can display opposite effects in responsive cells. Thus, while T3 enhances 3-methylcholanthrene induced transformation of C3H/10T1/2 cells by increasing K-*ras* levels (54), the hormone inhibits transformation of NIH-3T3 fibroblasts by oncogenic H-*ras* (55). Humans and experimental studies show that in contrast with the well-accepted role of TRβ as a tumor suppressor (56–59) TRα can have oncogenic effects *in vivo* (53, 60). The finding that T3 induces proliferation of immortalized macrophages agrees with this role of TRα, as these cells express this receptor and virtually no detectable TRβ.

The decreased number of immortal macrophages cultured in the absence of thyroid hormones could result from reduced proliferation and/or from increased cell death. Both mechanisms appear to contribute, as manifested by an increase in the number of apoptotic cells found in the absence of T3, but the major effect has to be attributed to the interruption in cell cycle progression, as the percentage of dead or apoptotic cells is low (approximately 10%) in comparison with the important increases in cell number observed in T3-treated cells. The potential target of the J2 virus is a M-CSF dependent cell, presumably a committed progenitor to the macrophage lineage (17) and M-CSF is the major and specific growth factor for this cell type. Accordingly, M-CSF also induced proliferation of immortalized macrophages cultured in depleted medium and had an additive effect with T3. That T3 might act, in part, by an autocrine mechanism is suggested by the finding that T3 increased *M-CSF* transcripts. Opposite to M-CSF, GM-CSF, a factor that stimulates macrophage production by bone marrow progenitor cells (61), did not induce proliferation of immortalized macrophages grown in depleted medium, but rather had an inhibitory effect on cell growth. Indeed, without decreasing cell viability, GM-CSF reduced the number of cells in the absence of T3, and was able to revert to a significant extent the stimulatory effect of the hormone and/or M-CSF. GM-CSF has also a clear inhibitory effect in the proliferation of immortalized macrophages grown in complete medium in the presence of endogenous hormone, suggesting that induction of *GM-CSF* transcripts by T3 could constitute a negative feed-back mechanism limiting the hormone proliferative effects.

Consistent with the effect of T3 in maintaining the growth of immortalized macrophages, the hormone orchestrates the activity of key signaling pathways with anabolic and proliferative functions, while inhibiting others with opposite effects. Thus, a higher ERK1/2 activation is found in T3-treated immortalized macrophages than in the untreated cells. The ERK1/2 pathway is stimulated by different growth factors and acts downstream of the *ras* and *raf* oncogenes, playing a key role in proliferating cells. In addition, this signaling pathway has been shown to regulate immune responses in macrophages (43, 62). In contrast, T3 inactivates the MAPK p38α. This is congruent with the function of this kinase that negatively regulates cell cycle progression at both the G1/S and G2/M transitions and induction of apoptosis by cellular stresses (63). The phosphatidylinositol-3-kinase (PI3K)/AKT pathway activates multiple downstream kinases that regulate many aspects of cellular activity including viability, metabolism and proliferation of different cell types including macrophages (64, 65) and is also induced by T3 in immortalized macrophages. AKT is in turn a critical activator of mTORC1, other crucial signaling pathway. mTORC1 coordinates the synthesis of macro-molecules needed in growing and proliferating cells (45), stimulating protein translation and ribosome biosynthesis, lipid homeostasis and synthesis of nucleotides providing the building blocks for cell growth. T3 induced the phosphorylation of the ribosomal protein S6, a crucial target of this pathway for protein synthesis. In contrast with AKT, AMPK stimulates catabolic pathways, while switching off energy-consuming processes such as biosynthesis and cell-cycle progression (46). Consistent with this role, although total levels of AMPK are increased by T3, its phosphorylation is strongly inhibited. This inhibition may be a consequence of AKT activation by the hormone, since AMPK is subjected to an inhibitory phosphorylation by different kinases including AKT (66), on a region different from Thr172 responsible for its activation. This inhibition may constitute a negative regulatory mechanism to switch off AMPK signaling favoring proliferation (67). In addition, AMPK causes an allosteric inhibition of mTORC1 (68) and therefore inhibition of AMPK activity by T3 should contribute to the maintenance of mTORC1 signaling.

Together with AKT and AMPK, Sirtuin-1 is a master regulator of metabolism, considered essential for adaptive responses (69, 70). In the metabolic effects of the thyroid hormones appear to be involved the regulation of Sirtuin-1 and there is a significant overlap between the actions of the thyroid hormones and this enzyme in the regulation of metabolic processes (71, 72). In immortalized macrophages T3 treatment increased Sirtuin-1 levels and, as expected, reduced p65 acetylation. Deacetylation by Sirtuin-1 leads to inactivation of p65, thus limiting the activity of the NF-κB pathway and the expression of NF-kB-dependent genes (48). We have previously shown that the thyroid hormones can antagonize NF-κB activation *in vivo* (34, 73, 74) and it is likely that p65 deacetylation might be involved in this regulation.

In contrast with the proliferative actions of T3 on immortalized macrophages, we have not been able to detect a clear effect of the hormone on the generation of primary macrophages. Although bone marrow cells cannot differentiate normally in serum depleted of thyroid hormones, T3 does not reverse this situation, suggesting that other still unidentified lipophilic factors, which are removed together with the thyroid hormones by the resin, are crucial for M-CSF-dependent differentiation of macrophage precursor cells. It had been reported that very high supra-physiological concentrations of T3 reduce the number and differentiation of bone marrow derived monocytes into unpolarized macrophages (28). However, our experiments with a more physiological hormone concentration do not show significant effects of T3 on macrophage differentiation in complete medium. Furthermore, after transfer of primary differentiated macrophages to depleted medium, treatment with the hormone for 48 h had not major effects on cell number, macrophage marker expression or transcript levels of polarization markers. Of the different signaling pathways analyzed, activation of ERK1/2, AKT, S6 and Sirtuin-1 by T3 was only observed in the cell line and appear to be related to the immortalization process rather than to the modulation of normal macrophage functions by the hormone. However, p38 and AMPK phosphorylation were reduced by T3 both in immortalized and primary macrophages. Down-regulation of AMPK activity has been shown to be essential in mediating the metabolic effects of the thyroid hormones (75) and we have previously shown in pituitary cells that T3 down-regulates p38 activation by a mechanism that involves the induction of the MAPK phosphatase DUSP1, and that this results in a reduction of the response to TNFα and NF-κB activity (74). The importance of regulation of these enzymes by T3 on macrophage functions requires further investigation.

The changes of signaling pathways activity in the T3-treated immortal macrophages correlated with important metabolomic alterations. The observed changes suggested an increased glycolysis in T3-treated cells with higher levels of lactate and a more efficient energy production with higher creatine levels. An increased glycolytic flux, typical of rapidly proliferating cells and inflammatory cells, promotes the accumulation of lactate (76). This is compatible with increased AKT and mTORC1 that increase glycolysis in macrophages (45) and with the reduced AMPK inactivation since this kinase promotes oxidative metabolism rather than glycolysis (46). In addition, succinate, a tricarboxylic acid cycle (TCA) intermediate, as well as glutamate, also accumulated in T3-treated cells. Succinate can accumulate as a consequence of a break-point in the Krebs cycle (11), which is also a typical feature of pro-inflammatory macrophages as a result of Krebs cycle interruption and OXPHOS suppression (6, 8, 11). mTORC1 has been also linked to glutamine metabolism through its control of glutamine hydrolysis to glutamate, the rate-limiting step in glutamine utilization, which can be converted to succinate as part of the Krebs cycle (9, 77). Interestingly, NAD^+^ levels also increased after T3 treatment, in concordance with Sirtuin-1 activation, while acetate that generates acetyl-CoA, of direct relevance to immunity and cell growth driving covalent modifications of chromatin proteins to alter the epigenome (78), was reduced by the hormone. The levels of formate, a one-carbon metabolite used for purine nucleotide biosynthesis, was reduced after incubation with T3, while the amino acid glycine, also involved in one‐carbon metabolism, which has recently been shown to regulate inflammatory responses (79) was increased. The amounts of other aminoacids, including taurine, valine, isoleucine and leucine, were reduced after T3 treatment, probably reflecting a higher utilization for protein synthesis consistent with the proliferative effects of the hormone. These changes were not observed in primary macrophages, in which cell proliferation was not induced by T3. The molecular mechanism/s responsible for the metabolic changes caused by T3 in immortal macrophages have not been identified in this study. However, the finding that the metabolic profile in T3-treated cells is compatible with that of rapidly proliferating cells, suggests that stimulation of ERK and AKT signaling pathways, as well as reduced activity of p38 and AMPK, could participate in this regulation.

Macrophages also utilize lipids for energy production. T3 increased free cholesterol levels in immortalized macrophages. Cholesterol provides structure and fluidity to the cell membranes and high levels of cholesterol in macrophages can lead to accumulation of lipid droplets and lipid raft formation, which is essential for pro-inflammatory signaling, demonstrating that cholesterol metabolism is a key biosynthetic pathway in macrophages (80). Sphingomyelin, other lipid metabolite increased by T3, is the most abundant sphingolipid, also associated with lipid rafts, and its interaction with cholesterol regulates cell signaling pathways and apoptosis (81). Both have been shown to increase upon classical macrophage activation (82, 83). The balance between FA oxidation and FA synthesis may be essential to macrophage function. FA oxidation is necessary to support the functions of alternatively activated macrophages (10) and overall levels of free FA are diminished in inflammatory compared to resting macrophages (84). Thus, the overall metabolic changes produced by T3 in immortalized macrophages are reminiscent of those caused by pro-inflammatory stimuli, and this coincides with increased expression of genes such as *TNF*α or *HIF-1*α. However, T3 also increased *Arginase 1* mRNA, suggesting a complex effect of the hormone I-BMDM function, not directly related to a typical polarized phenotype. In fact, metabolic changes observed in T3-treated immortal macrophages can also be interpreted as being characteristic of rapidly dividing cells providing the anabolic changes and metabolites needed for making new cells. This is strongly suggested by the finding that T3 had little metabolic effects in primary macrophages.

PUFAs were the only class of lipid metabolites induced by T3 in both immortal and primary macrophages. These compounds affect macrophage functions through the production of bioactive lipid mediators, through changes of cell membrane properties or by acting as ligands for membrane and nuclear receptors (85). PUFAs, are involved in inflammatory processes and their resolution (86). PUFA levels in macrophages are subject to temporal changes during the inflammatory response. Macrophage content of PUFAs is rapidly decreased following pro-inflammatory stimuli, correlating with downregulation of genes involved in PUFA synthesis, followed by a late phase response associated with increased intracellular PUFA levels, as well as induction of the genes responsible for their generation (82, 83). Therefore, control of PUFA levels by T3 might regulate the macrophage response to inflammatory stimuli.

Transcriptomic analysis confirmed that T3 has a crucial role in the proliferation of immortal macrophages stimulating expression of gene sets involved in protein synthesis and cell cycle progression. Moreover, it also confirmed the regulation of genes involved in the observed metabolic changes, with an increase in glycolysis/gluconeogenesis and a reduction in cholesterol metabolism that could lead to its accumulation. The transcriptomic changes were also less marked in primary macrophages than in the immortalized cells, with a lower number of regulated transcripts and weaker changes. Not only the changes were weaker, but T3 modified different pathways and processes with only a small number of common transcripts being regulated with the same tendency in immortal and primary macrophages. In fact, while changes in expression of genes involved in cell proliferation were predominant in immortal macrophages, in primary macrophages the most enriched processes after T3 treatment were related with transcriptional regulation, corresponding with the actions of TRα as a transcription factor that recruits both coactivators and corepressors to stimulate or repress gene expression (19).

It has been reported that immortal macrophages and primary macrophages display similar metabolic and functional features upon cytokine stimulation (18). We observed that, indeed, in both types of cells T3 reduced STAT-1 phosphorylation in response to IFNγ, the key regulator of macrophage activation. Besides the canonical pathway, IFNγ can also activate ERK1/2 in some cells (87). This was observed in primary macrophages and T3 also reduced this activation. These results extend our previous observations that the thyroid hormone can antagonize IL-6 signalling leading to reduced STAT-3 transcriptional activity (34). Besides decreasing STAT-1 phosphorylation in response to IFNγ, T3 reduced phosphorylation of the upstream activator JAK2, suggesting that T3 could be a general inhibitor of the response to different factors signalling through the JAK/STAT pathway. JAK/STAT signaling begins with the binding of cytokines to the cytokine receptors. Receptor oligomerization allows the transphosphorylation of JAKs that then phosphorylate the receptors creating a docking site for STATs (88). Any of these step/s could be regulated by T3 and be responsible for the observed antagonism.

In conclusion, T3 is crucial for the growth on oncogene-immortalized macrophages, but had little metabolic and transcriptomic effects in the resting unpolarized normal counterparts. As T3 could regulate the response to pro-inflammatory stimuli (26, 28, 29), future studies are needed to underscore a potential function of the hormone on metabolism and global gene expression after cytokine stimulation.

## Data availability statement

The datasets presented in this study can be found in online repositories. The names of the repository/repositories and accession number(s) can be found below:

https://www.ncbi.nlm.nih.gov/, GSE200718.

## Ethics statement

The animal study was reviewed and approved by Ethics Committee of the Consejo Superior de Investigaciones Científicas and Comunidad de Madrid.

## Author contributions

AA and SA conceptualized and designed the study. IL-M and DR-M conducted experiments. AA, SA and AC obtained funds. JR and AC analyzed data. AA wrote the manuscript and SA and AC critically revised the manuscript. All authors contributed to the article and approved the submitted version.

## Funding

This work has been funded by grants SAF2017-83289-R and PID2020-116146RB-I00 to SA and AA, PID2019-104284RB-I00/AEI/10.13039/501100011033 to AC and SAF2017-90604REDT to AA and AC from Ministerio de Ciencia Innovación y Universidades; by grant B2017/BMD-3724 to SA and AA from the Comunidad de Madrid; and by CIBERONC CB/16/00228 to AA from the Instituto de Salud Carlos III. The cost of this publication has been paid in part by FEDER Funds.

## Conflict of interest

The authors declare that the research was conducted in the absence of any commercial or financial relationships that could be construed as a potential conflict of interest.

## Publisher’s note

All claims expressed in this article are solely those of the authors and do not necessarily represent those of their affiliated organizations, or those of the publisher, the editors and the reviewers. Any product that may be evaluated in this article, or claim that may be made by its manufacturer, is not guaranteed or endorsed by the publisher.
