# Supplementary material for: Regulation of metabolic and transcriptional responses by the thyroid hormone in cellular models of murine macrophages
Source: Front Immunol. 2022 Jul 22;13:923727. doi: 10.3389/fimmu.2022.923727 (PMC9353060; doi:10.3389/fimmu.2022.923727)
Supplement: Supplementary file 1 [file DataSheet_1.pdf]

Supplementary Material

1 SUPPLEMENTARY DATA

Primers used for Quantitative real-time PCR

	Forward Primer	Reverse Primer
**TRα**	CTGACCTCCGCATGATCGG	GGTGGGGCACTCGACTTTC
**TRβ**	GGACAAGCACCCATCGTGAAT	CTCTGGTAATTGCTGGTGTGAT
**ARG 1**	TCATTTGGGTGGATGCTCACAC	GAGAATCCTGGTACATCTGGGAA
**EGR2**	AACGGAGTGGCGGGAGAT	ATGGGAGCGAAGCTACTCGGAT
**YM1**	GGGCATACCTTTATCCTGAG	CCACTGAAGTCATCCATGTC
**IL1b**	TGGACCTTCCAGGATGAGGACA	GTTCATCTCGGAGCCTGTAGTG
**IL12a**	ACGAGAGTTGCCTGGCTACTAG	CCTCATAGATGCTACCAAGGCAC
**IL12b**	TTGAACTGGCGTTGGAAGCACG	CCACCTGTGAGTTCTTCAAAGGC
**GM-CSF**	TCGTCTCTAACGAGTTCTCCTT	CGTAGACCCTGCTCGAATATCT
**M-CSF**	ATGAGCAGGAGTATTGCCAAGG	TCCATTCCCAATCATGTGGCTA
**TNFα**	CGATCACCCCGAAGTTCAGTA	GGTGCCTATGTCTCAGCCTCTT
**HIF-1**	GGGGAGGACGATGAACATCAA	GGGTGGTTTCTTGTACCCACA
**AMPK**	GTCAAAGCCGACCCAATGATA	CGTACACGCAAATAATAGGGGTT
**Sirt-1**	TGATTGGCACCGATCCTCG	CCACAGCGTCATATCATCCAG

List of antibodies used

ANTIBODY	CATEGORY	DILUTION	SUPPLIER	Cat. number
**Phospho-ERK**	Mouse monoclonal	1:1000	Santa Cruz Biotechnology	sc-7383
**ERK2**	Rabbit polyclonal	1:20000	Santa Cruz Biotechnology	sc-154
**Phospho-p38 (Thr180/Tyr182)**	Rabbit polyclonal	1:1000	Cell Signaling	9211
**p38**	Rabbit polyclonal	1:20000	Santa Cruz Biotechnology	sc-728
**Phospho-Akt1/2/3 (Ser 473)**	Rabbit polyclonal	1:1000	Santa Cruz Biotechnology	sc-7985-R
**AKT3 + AKT1**	Rabbit monoclonal	1:1000	Abcam	ab32038
**Phospho-p70 S6 kinase (Thr 389)**	Rabbit polyclonal	1:1000	Santa Cruz Biotechnology	sc-11759-R
**p70 S6 kinase**	Rabbit polyclonal	1:1000	Santa Cruz Biotechnology	sc-9027
**Phospho-S6 (Ser240/244)**	Rabbit polyclonal	1:1000	Cell Signaling	2215
**S6 Ribosomal Protein**	Rabbit monoclonal	1:1000	Cell Signaling	2217
**Phospho-AMPKα (T172)**	Rabbit monoclonal	1:1000	Cell Signaling	2535
**AMPKα**	Rabbit polyclonal	1:1000	Cell Signaling	2532
**Sirtuin 1**	Rabbit polyclonal	1:1000	Sigma-Aldrich	07-131
**NF-kB p65 (acetyl K310)**	Rabbit polyclonal	1:500	Abcam	ab19870
**Phospho-p65 (Ser536)**	Rabbit monoclonal	1:1000	Cell Signaling	3033
**IKBα**	Rabbit polyclonal	1:1000	Santa Cruz Biotechnology	sc-371
**p65**	Rabbit monoclonal	1:1000	Cell Signaling	8242
**Actin**	Goat polyclonal	1:10000	Santa Cruz Biotechnology	sc-1615
**Phospho-Jak2 (Tyr1007/1008)**	Rabbit polyclonal	1:1000	Cell Signaling	3771

**Phospho-Stat1 (Tyr701)**	Rabbit monoclonal	1:1000	Cell Signaling	9167
**Stat1**	Rabbit Polyclonal	1:1000	Cell Signaling	9172

2. SUPPLEMENTARY FIGURES

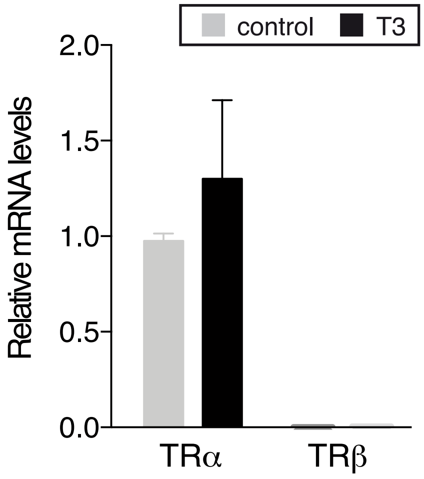

Supplementary Figure 1. TRs expression in immortalized macrophages. mRNA levels of TRα and TRβ were measured by quantitative PCR in I-BMDM treated in the absence (control) and presence of 10 nM T3 for 48 h in thyroid hormone-depleted medium (n=6). Data are means±SD and are expressed relative to the values of TRα mRNA in the untreated cells.

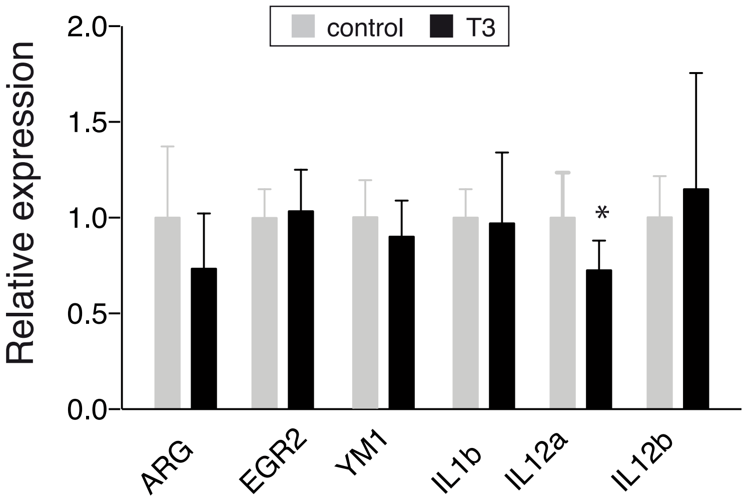

Supplementary Figure 2. Effect of T3 on expression of polarization markers in bone marrow derived macrophages. Levels of the indicated transcripts were measured by quantitative PCR in primary bone marrow derived macrophages (P-BMDM) differentiated in complete medium in the absence (control) and presence of 10 nM T3. (n=6-9). Data are means±SD and are expressed relative to the values of the control cells. *$p < 0.05$.

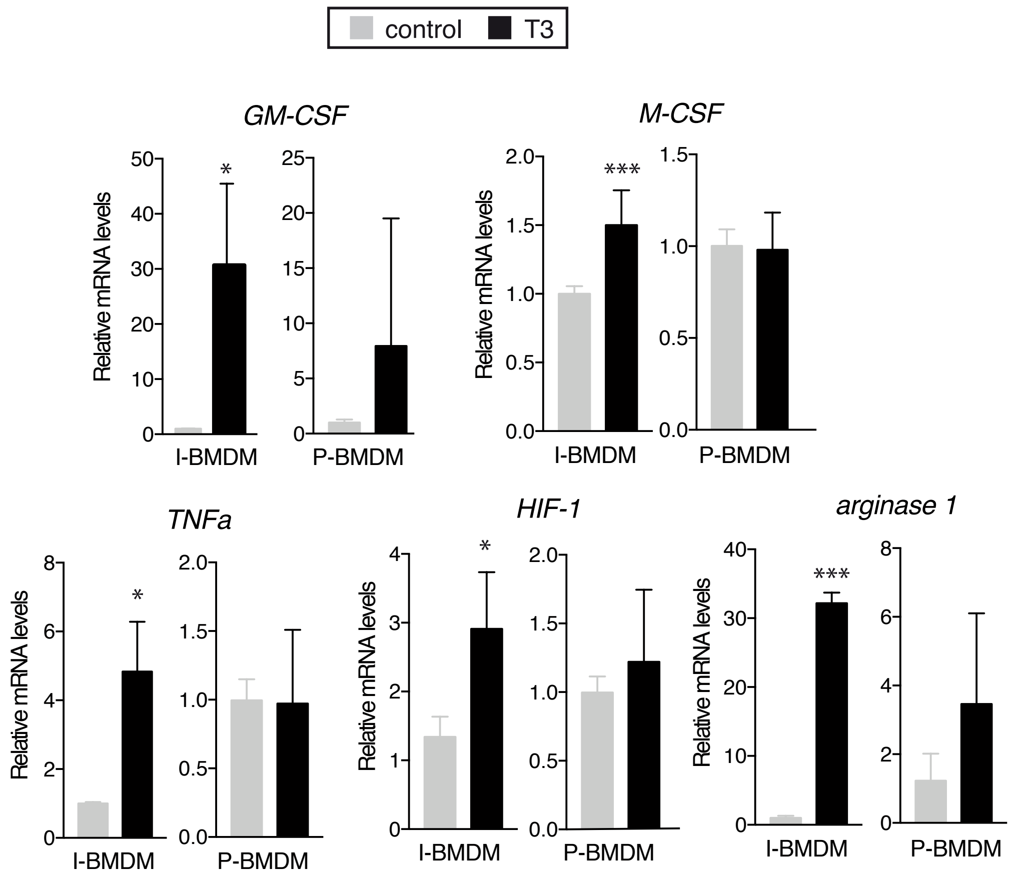

Supplementary Figure 3. Effect of T3 on gene expression in immortalized and primary bone marrow derived macrophages. Primary macrophage (P-BMBM) were differentiated for 5 days in complete medium, shifted to thyroid hormone depleted medium and incubated in this medium for 48 h in the absence and presence of T3 (n=9). Immortalized macrophages (I-BMDM) were treated in a similar way (n=6). Levels of the indicated transcripts were measured by quantitative real-time PCR. Data are means±SD and are expressed relative to the values obtained in the corresponding control untreated cells. *p < 0.05, ***p < 0.001.

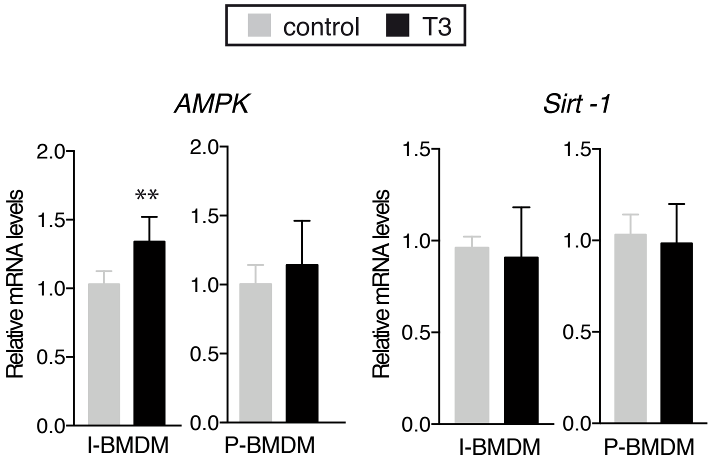

Supplementary Figure 4. Effect of T3 on AMPKα and Sirtuin-1 gene expression in immortalized and primary bone marrow derived macrophages. Primary macrophages (P-BMBM) were differentiated for 5 days in complete medium, shifted to thyroid hormone depleted medium and incubated in this medium in the absence and presence of T3 for 48 h (n=6). Immortalized macrophages (I-BMDM) were treated in a similar way (n=6). mRNA levels are represented as means±SD and are expressed relative to the values obtained in the corresponding control untreated cells. *p < 0.05, ***p < 0.001.

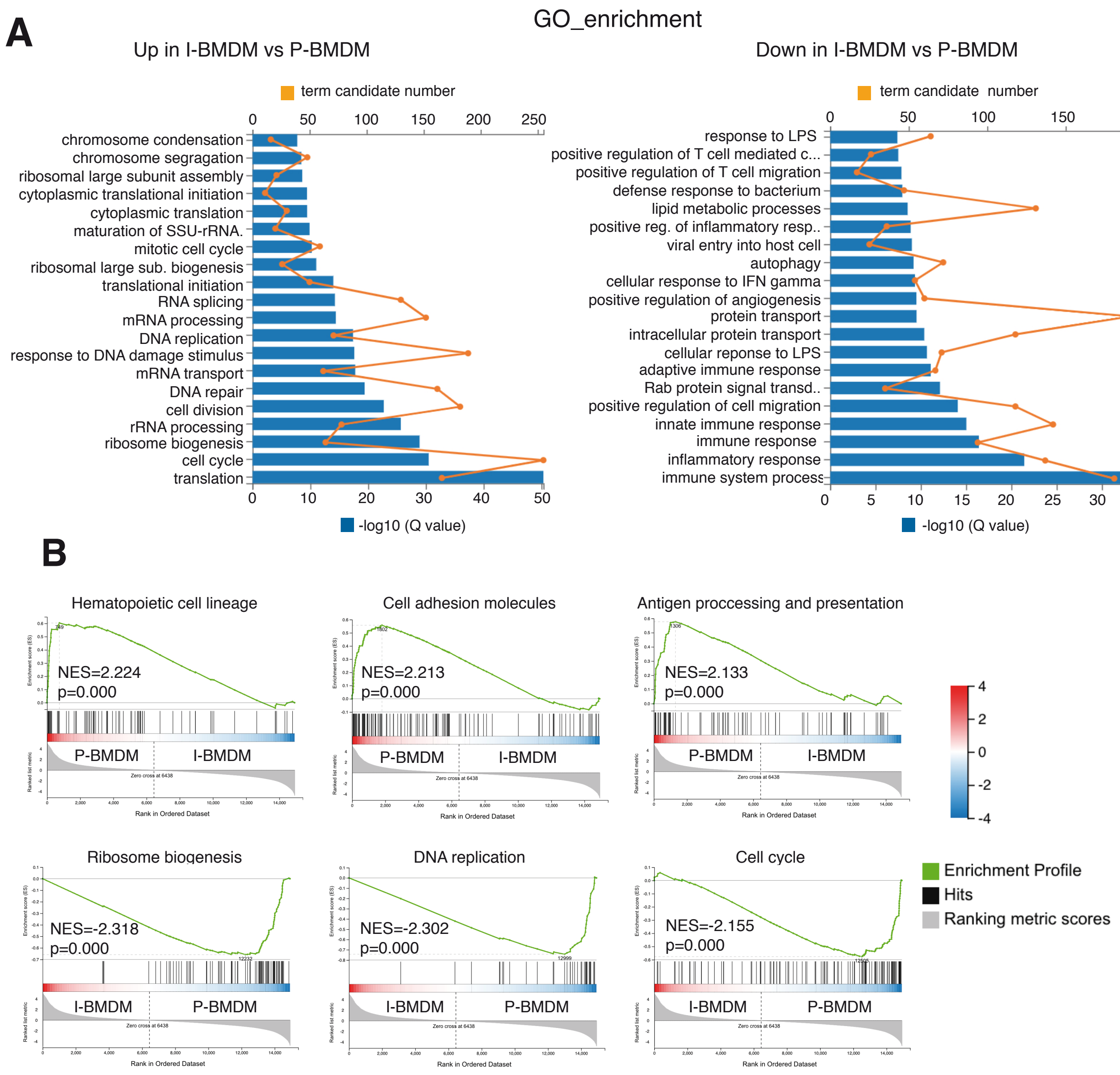

Supplementary Figure 5. Transcriptomic changes between immortalized and normal bone marrow derived macrophages. **(A)** Gene ontology (GO) categories with significant enrichment (left panel) or reduction (right panel) in the transcripts of immortalized macrophages (I-BMDM) vs primary cultures of macrophages (P-BMDM). Both the term candidate transcript number (in orange) and the Q value (in blue bars) are represented. **(B)** Enrichment plots from Gene Set Enrichment analysis (GSEA) based in KEEG pathway database showing the top over-represented hallmarks in up-regulated and down-regulated genes I-BMDM vs P-BMDM. The normalized enrichment scores (NES) as well as the nominal p values are shown.
